# Monitoring the production of reactive oxygen species in experimental melanoma

**Published:** 2013-09-25

**Authors:** AV Lazescu, MI Gruia, R Anghel, D Glavan

**Affiliations:** * Oncology Department, “Carol Davila" University of Medicine and Pharmacy, Bucharest “Prof. Dr. Alexandru Trestioreanu" Institute of Oncology, Bucharest; ** “Prof. Dr. Alexandru Trestioreanu" Institute of Oncology, Bucharest

**Keywords:** melanoma, murine models, oxidative stress, marker

## Abstract

Abstract Hypothesis: Melanoma is one of the most aggressive forms of skin cancer characterized by malignant proliferation of melanocytes. The role played by reactive oxygen species and free radicals in the pathology of melanoma in humans is widely accepted today. Objective: This paper aims to characterize some types of malignant melanoma obtained experimentally by the inoculation of reference cells for the creation of models and the identification of oxidative stress markers usable for monitoring tumor growth and development. Methods and results: Mice C57Bl/6. Reference cell lines B16, F1, F10. Inoculation of cells was performed in the upper right flank. Tumors were characterized both anatomically and morphologically. For the biochemical characterization of the oxidative stress, tests were performed to determine lipid peroxides, total albumin thiol groups and total antioxidant response. Tumor volume was measured in dynamic. The fastest development has been observed in type B melanoma. For the F and F10 types, the curves profiles are the same. The results indicate an increase of lipid peroxidation reaction in dynamic tumor evolution, suggesting the malignant associated transformations. Discussion: These data demonstrate that an alteration of the antioxidant pattern can be detected in the serum of the experimental animals with melanoma, possibly related to the disease status and progression. Our results can be useful in monitoring the tumor evolution and also to highlight the prolonged damage which actions on the normal cells, suggesting the combination of the classical treatments with an adjuvant antioxidant treatment.

## Introduction

Melanoma is one of the most aggressive forms of skin cancer characterized by malignant proliferation of melanocytes (cells originating from the neural crest) [**1**]. Aggression is based on several mechanisms: increased capacity of invasion and metastasis, expressed since the early stages of tumor progression; remarkable genotypic and phenotypic heterogeneity, increased resistance to conventional therapeutic means (immunotherapy, radiotherapy, chemotherapy) [**2**]. Current statistics say that if diagnosed in early stages of evolution melanoma can be successfully treated, while metastatic melanoma is fatal in almost all cases [**3**]. Resistance to therapy is due to this remarkable cellular heterogeneity, characterized by: karyotype variability, the growth rate, antigenicity and immunogenicity, the ability of invasion and metastasis, drug sensitivity to cytotoxic agents [**4**].

 Oxygen is a paradoxical element, because it is equally essential for the life of aerobic organisms but also toxic, destructive through its active metabolites. In normal state, the oxygen molecule is stable and therefore less reactive, combines directly with organic compounds (spontaneous combustion) and allows oxidation of substances with the energetic role, possibly adenosine-triphosphate (ATP) synthesis in the respiratory chain [**5**]. During intracellular metabolism, oxygen can undergo activation (the gain of electrons or energy gain) with the formation of highly reactive chemical species [**6**].

Therefore, the cell is continuously exposed to the action of cytotoxic reactive oxygen species. They are a potential threat to cellular integrity because each reactive species will seek an electron in the environment to stabilize itself, thus generating chain reactions. The role of reactive oxygen species and free radicals in human pathology is widely accepted today [**7**]. The primary effect of oxidative stress is localized and reversible, depending on the intensity and level of antioxidants [**8**]. This primary effect can last for years, may be offset or extended. The transition to side effect marks the irreversible phase. If in the primary stage the symptoms are minor, the switch to the side effect is accompanied by the appearance of clinical manifestations due to cell lysis, altered function, low resistance to effort, or infection [**9**]. Associated severe illness becomes apparent. Reactive oxygen species and especially the radicals attack macromolecules which are essential for life, generating dysfunctions in cell metabolism, thus explaining oxygen toxicity [**10**].

 Induced oxidative degradation reactions can be a primary cause of a significant number of pathologies, manifested by altered signaling and transmitting information systems at intra and extracellular levels [**11**], and were therefore called “informational pathologies" [**12**]. The diseases included in this category are alcoholism, atherosclerosis, bronchitis, emphysema, cancer, alcoholic cirrhosis, diabetes, ischemia/reperfusion, aging [**13**]. The degenerative reactions initiated by the reactive oxygen species can induce complications or may be a consequence of cell injury caused by other agents; therefore, the relationship between metabolites of oxygen and the disease is dual, of cause ↔ effect type [**14**]. The answer to this equation is still uncertain: active metabolites of oxygen present in excess are a cause or a consequence of installing a form of disease mediated by them? [**15**].

### Purpose 

 This paper aims to characterize some types of malignant melanoma obtained experimentally by the inoculation of reference cells in order to obtain models and to identify the oxidative stress markers usable for monitoring tumor growth and development. 

## Materials and methods 

 We used C57Bl/6 mice and B16, F1, F10 reference cell lines. 

 Inoculation of cells was performed in the upper right flank. The melanocytes were grown in a humidified atmosphere at 37°C under 5% CO2.

 The study respected Animals and Treatment Protocols. Anesthesia of animals was done with Acepromazine 1%, 2.5 mg/body weight and with Ketamine 10%, 75 mg/body weight. This combination of two drugs was chosen to provide the relaxation for a minimum of 2 hours. 

 Tumors were characterized both anatomically and morphologically.

 For the biochemical characterization of the oxidative stress, tests were performed to determine lipid peroxides, total albumin thiol groups and total antioxidant response.

 All tests were performed in a consecutive tumor growth dynamics in several passages in serum. Lipid peroxides were assessed by measuring the concentration of malon dialdehyde, the end product of lipid hydro peroxides degradation derived from biochemical reactions. The method is based on forming a red colored adduct (malondialdehyde thiobarbituric acid-MDA-TBA2) which has a peak of absorption at 532 nm.

 SH-albumin groups were determined based on the reaction with acid 5.5 '-dithio-bis (2-nitrobenzoic) Ellman reagent, DTNB noted.

 In order to determine the total antioxidant level, the ability of serum to reduce iron was used. At low pH complex Felll - tripiridil-s-triazine (Felll-TPTZ) is reduced to the ferrous form with a complex formation that generates a new intense blue color, measurable, with a peak of absorption at 593 nm. Any reaction with a positive redox potential condition mentioned may reduce Felll-TPTZ complex. Using an excess of Fe, the limiting factor of the complex and color formation Felll-TPTZ, represents the reducing ability of the sample. The reaction measures the reduction of ferric ion complex 2,4,6-tri (2-pyridyl) -1,3,5-triazine (TPTZ) to a colored product.

## Results 

**Table 1:** Morphologic and functional features of experimentally obtained tumors

	liver	spleen	kidney	lung	other metastases
B	discolored, friable	enlarged, splenic follicles obvious	discolored, friable, congestive subcapsular areas	congestive bleeding areas macroscopic metastases	tracheobronhial enlarged lymph nodes
F10	discolored, friable	friable	discolored, friable	congestive gray areas macroscopically undetectable metastases	peritoneal metastases (unpigmentated, well vascularized)
F	discolored, friable	enlarged	discolored, friable	infiltrative aspect macroscopically undetectable metastases	Tracheobronhial enlarged, gray lymph nodes

**Chart 1:**
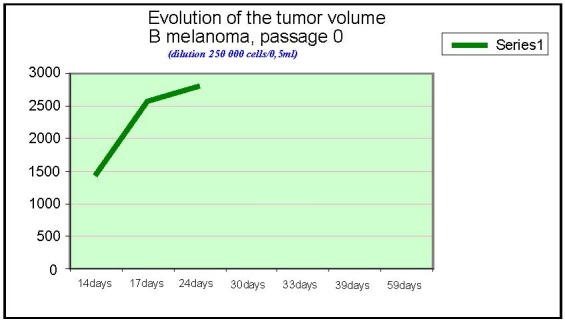
Evolution of tumor volume, B melanoma, passage 0

**Chart 2:**
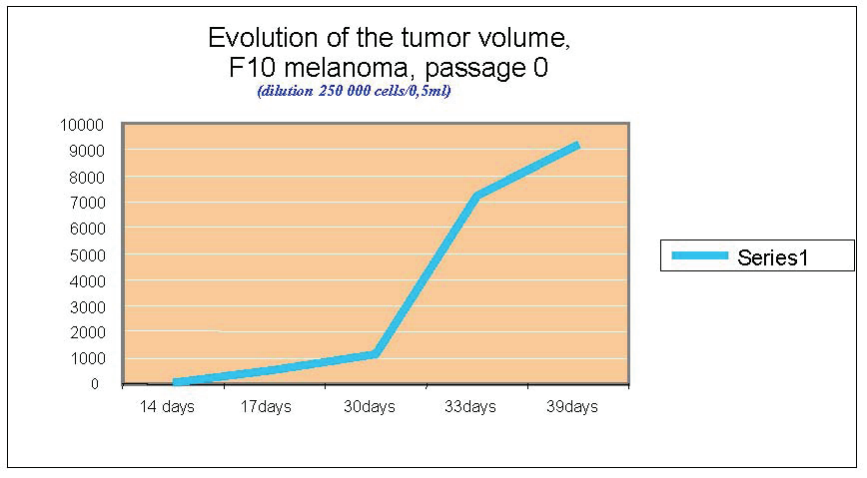
Evolution of tumor volume, F10 melanoma, passage 0

**Chart 3:**
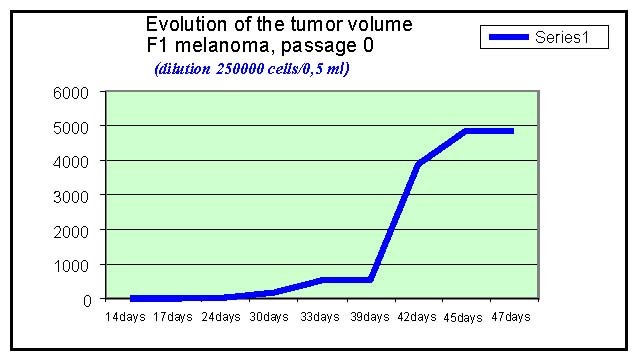
Evolution of tumor, F1 melanoma, passage 0

**Chart 4:**
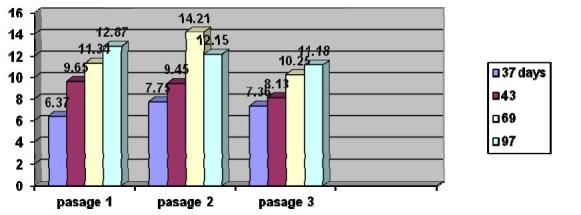
Reaction measured in serum lipid peroxidation

Tumor volume evolution 

 The tumor volume was measured in dynamics, the first signs of tumor appearance are noted 14 days after the graft, using the same number of cells in the inoculate. 

 The tumor volume was calculated by using the formula: 

Vtumoral = a x b ² x 0,52

Where a is the biggest tumor diameter, 

b is the small diameter 

 0,52 represent the skin depth 

As the graphics show, the fastest development was observed in B type melanoma. For F and F10 type, the curves have the same profile.

Biochemical characteristics of experimentally obtained tumors:

Lipid peroxidation values obtained in dynamic 

 The results indicate an increase of lipid peroxidation reaction in dynamic evolution of tumor, suggesting the malignant associated transformations. The second passage shows the same metabolic profile of lipid peroxidation reactions compared to the first passage. Distinctions are insignificant due to individual response in vivo. The metabolic profile looks the same after the third passage.

Determination of thiol groups

 The effect of active oxygen metabolites on the structural or enzymatic proteins determines their distortion. Most frequently, interactions occur with protein-thiol groups, which in turn may have dimerisation or oxidation with formation of disulphides, respectively sulphonic acid derivatives. The oxidation rate of thiols or proteins containing -SH groups (sulfhydryl groups) decreases in the following order: SH-hemoprotein > SH-oxyhemoglobin > simple thiols > SH-protein (serum albumin) [**16**].

 A decisive factor in this reaction, the oxidation of protein-SH groups by the reactive oxygen species, is the steric factor. Exposure of protein thiol groups is essential. This explains the increased sensitivity to oxidation of denatured proteins (this property is used as an effective method of identifying the content in thiol groups and to identify the secondary and tertiary structure of proteins).

The oxidative degradation inhibits enzyme activity due to the changed catalytic active center (due to oxidation of thiol groups or heme, but also by oxidative degradation of the structure of aromatic amino acids) [**17**]. Structural proteins are inactivated due to changes induced by polymerization reactions, branching or split polypeptide chains induced by reactive oxygen species.

**Chart 5:**
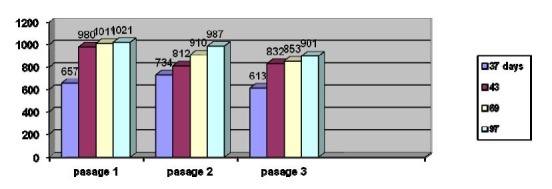
Albumin thiols evolution measured in dynamic of serial passages

**Chart 6:**
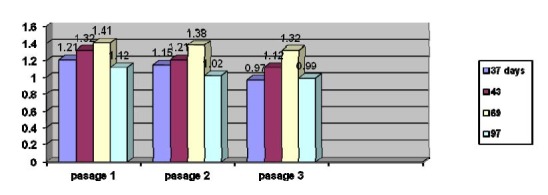
Lipid peroxidation according to tumor evolution

No significant changes are recorded from a passage to another, and variations are caused by an increase in intensity of oxidation reaction due to increasing the concentration of protein-SH groups free.

Determination of the total antioxidant 

 A biological antioxidant may be defined as any substance present in lower concentration than the oxidable substrate, which significantly reduces or prevents oxidation of the substrate. This definition covers each team member of the antioxidant defense (antioxidant enzymes primary, secondary, lipophilic antioxidants, water-soluble plasma proteins, etc.) Simply put, non-enzymatic antioxidants such as ascorbic acid, tocopherol, carotene, Fe carrier proteins, can be described as reduction and inactivated oxidants participating in redox reactions in which reactive species are reduced due to the oxidation of another [**18**]. In this context, the antioxidant power can be reported by analogy to the reducing ability.

 Several tests measure the effect of non-enzymatic antioxidant defense in biological fluids and can be used to determine the resistance index of oxidative attack. Most tests used to measure the total antioxidant power of plasma currently measure the ability of plasma to resist the oxidative effects of reactive species generated in the reaction mixtures. Antioxidants decrease is marked by a change in the signal such as oxygen utilization rate, or chemiluminescence. Measurement of these signals requires specialized equipment, and these tests are time consuming and need special technique, making clinical evaluative studies difficult to perform [**19**].

 The technique that we submit to your attention is the principle method using colorimetric tests related reduction in redox reactions, using a slightly reduced oxidant in stoichiometric excess.

 Oxidable + Antioxidant Reduced + Antioxidant substrate -> substrate linked to reduced substrate

The obtained data suggest the activation of antioxidant defense systems by limiting the intensity of reaction measured and its profile when installing the oxidative stress.

## Discussion

The macroscopically observed results obtained in this experiment do not emphasize significant changes between the 3 cell types in vivo inoculations in experimental animals. It is possible that, in vivo, the cells will react differently than in a culture, a lot of immune protection systems being involved, which can be overtaken at one moment and thus allowing the installation and development of melanoma tumors [**20**].

The subcutaneous transplantation, which is the most common way of grafting, was performed because tumors obtained in this manner can be easily observed and measured. Tumor growth is the consequence of increasing the number of tumor cells and occurs when tumor cell proliferation rate exceeds the loss [**21**]. In this case, there is a small lag period that allows growth and tumor tracking, which makes this experimental tumor type offer several advantages for experimental models.

 These results demonstrate that an alteration of the antioxidant pattern can be detected in the serum of experimental animals with melanoma, possibly related to the disease status and progression. Free oxygen reactive species production is modified during tumor development.

 Our results can be useful in monitoring the tumor evolution and also to highlight the prolonged damage which affects the normal cells, suggesting the combination of the classical treatments with an adjuvant antioxidant treatment. 
